# Appendicular Diverticulum and Colorectal Neoplasia: A Case Series and Literature Review

**DOI:** 10.7759/cureus.88254

**Published:** 2025-07-18

**Authors:** Martín Encalada, Igor Michalick, Marcelo Girundi, Rodrigo Cardoso

**Affiliations:** 1 General Surgery, São Francisco de Assis Hospital Foundation, Belo Horizonte, BRA; 2 Trauma and Oncologic Surgery, São Francisco de Assis Hospital Foundation, Belo Horizonte, BRA; 3 General and Bariatric Surgery, São Francisco de Assis Hospital Foundation, Belo Horizonte, BRA

**Keywords:** appendectomy variants, appendicular diverticulitis, appendicular diverticulosis, colo rectal cancer, right-sided diverticulitis

## Abstract

Appendicular diverticulosis is a rare condition often discovered incidentally and associated with an increased risk of complications such as perforation, bleeding, and neoplasia. We present three cases of appendicular diverticulosis with varying presentations: one associated with acute appendicitis and two identified incidentally during surgical procedures. After a thorough literature review to back up this report, 19 cases were found with this condition and similar characteristics: of 19 patients, the majority were aged 40-60 years, with a slight male predominance, and abdominal pain was the most common symptom. The primary diagnostic modality was abdominal computed tomography, with perforation observed in approximately 27% of cases and one instance of appendiceal adenocarcinoma identified. The potential for serious complications underscores the importance of early diagnosis and individualized management to prevent morbidity, given the higher likelihood of neoplasia and perforation in patients with this condition.

## Introduction

Appendicular diverticulosis (AD) is a rare and often overlooked pathological condition characterized by the presence of diverticula-saccular outpouchings of the mucosal or submucosal layers-within the appendix. These diverticula can be either congenital, involving all layers of the appendiceal wall (true diverticula), or acquired, typically consisting only of mucosa and submucosa herniating through a defect in the muscularis (false diverticula). The acquired form is more common and is generally associated with chronic inflammation or increased intraluminal pressure. While diverticulosis is more commonly encountered in the colon, particularly in the sigmoid region, AD remains exceptionally rare, with a reported prevalence ranging from 0.004% to 1.74% [1-4].

Unlike colonic diverticulosis, which often presents with benign or chronic symptoms, AD is more likely to result in serious complications, including inflammation, perforation, hemorrhage, and neoplasia. Notably, the perforation rate in AD has been reported to range from 5-10%, significantly higher than that of acute appendicitis [5].

Preoperative diagnosis of AD is uncommon due to its non-specific clinical and radiologic features, and in most cases, it is identified incidentally during surgery or through postoperative histopathological analysis. This diagnostic difficulty can delay appropriate management and increase the risk of adverse outcomes. Furthermore, AD has been associated with a markedly increased risk of neoplasia, including mucinous adenomas and carcinoid tumors, with neoplastic changes present in up to 26.94% of patients with AD, compared to only 1.28% in those without diverticula [6].

Given the potential for severe complications and its diagnostic challenge, timely recognition and appropriate surgical management of AD are essential.

The objectives of this article are to present three cases of AD identified during surgical procedures, to review the current literature on its clinical presentation, diagnostic challenges, and complications, and to highlight the importance of awareness and early intervention to improve patient outcomes.

## Case presentation

We present three distinct cases of patients with AD.

Case 1: A.E.G.L.

A 73-year-old male patient with a history of stroke in 2022, which resulted in motor sequelae, type 2 diabetes mellitus, and gout.

He was admitted to the emergency department in March 2024 with tight chest pain and an episode of vomiting, in addition to chronic melena. He reported pain for three months, with recent worsening. An upper digestive endoscopy was performed, without changes and/or active bleeding, and a partial colonoscopy revealed an endophytic and stenosing lesion in the descending colon, suggesting malignancy, later confirmed by histopathology as colonic adenocarcinoma.

The CT scan revealed a marked circumferential parietal thickening of the middle/proximal third of the transverse colon, close to the hepatic angle. No acute changes in the blood tests were seen. 

In May 2024, the patient underwent rectosigmoidectomy for treatment of adenocarcinoma, including lymphadenectomy and appendectomy, where appendiceal diverticula were found as an incidental finding, confirmed in the posterior biopsy result.

In the late postoperative period, he presented with abdominal distension and pain, requiring an exploratory laparotomy on the sixth postoperative day, which identified a perforation in the anastomosis.

**Figure 1:**
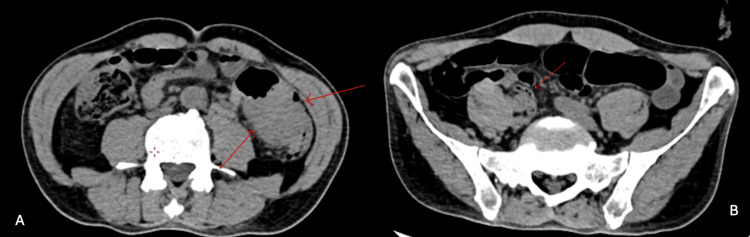
Abdominal CT - Case 1 Case 1. A: Tumor in the descending colon. B: Appendix. The CT reported marked circumferential parietal thickening of the middle/proximal third of the transverse colon, close to the hepatic angle. It didn't report appendicular diverticulosis or diverticulitis, only making the diagnosis of AD after the biopsy was studied.

**Figure 2:**
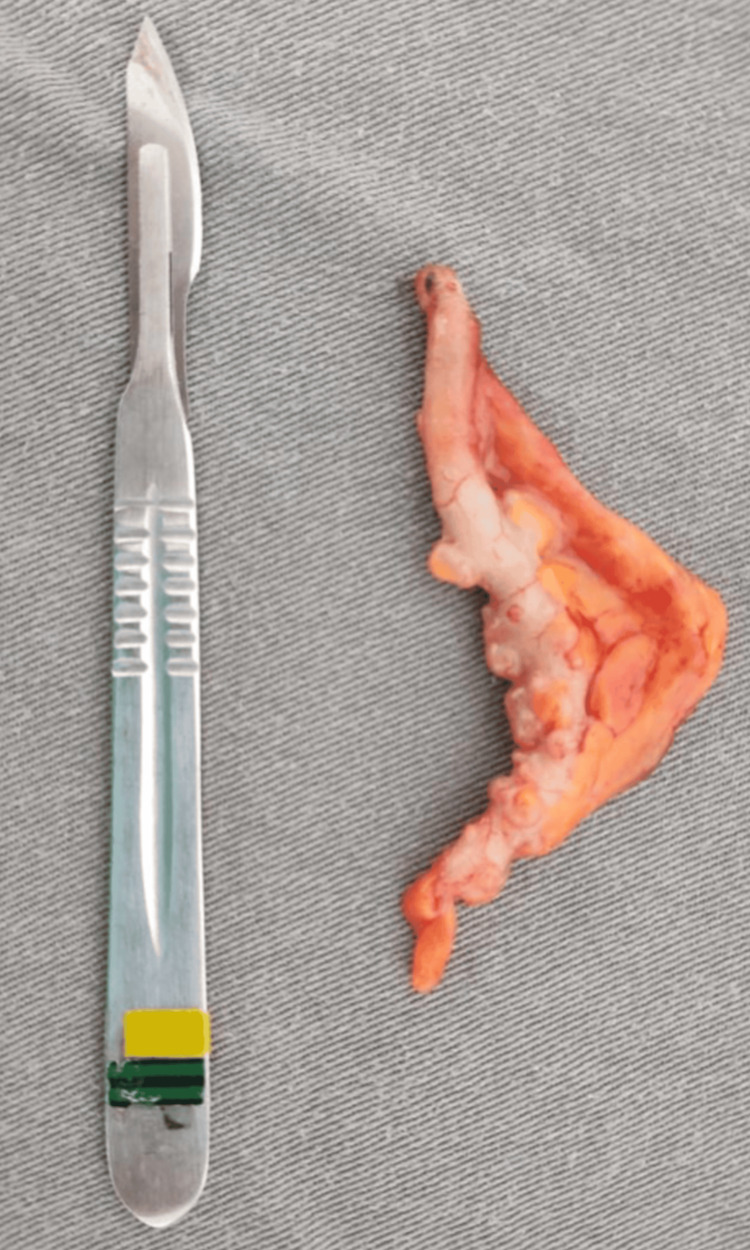
Appendectomy product - Case 1 Case 1. Appendiceal diverticula were seen along the entire length of the specimen, with no signs of inflammation, perforation, or neoplasia.

A colectomy and colostomy were performed as a corrective measure. The patient remained in the ICU for five days, using vasoactive amines, antibiotic therapy, sedation, and nutritional support via nasoenteric tube, with no evident improvement. Eleven days after the rectosigmoidectomy, the patient developed hypotension and cardiorespiratory arrest on June 9, 2024, and death was confirmed at 10:51 pm. Figure 1 and Figure 2 show the patient's CT and appendectomy product. The CT scan did not report AD or diverticulitis, only making the diagnosis of AD after the biopsy was studied.

Case 2: A.A.S.

A 64-year-old male with a history of silicosis, prediabetes, and smoking (52 pack-years), reported chronic abdominal pain that began in 2022, associated with constipation with thin, dark stools, in addition to a weight loss of 10 kg in the last two years. In March 2024, a colonoscopy identified a lesion in the splenic angle of the colon and polyps in the sigmoid colon, histopathologically confirmed as invasive adenocarcinoma and low-grade tubular adenoma, respectively.

In April 2024, computed tomography showed circumferential parietal thickening in the transverse colon, close to the hepatic flexure, with luminal reduction of the colon and bilateral lung masses, suggesting a neoplastic nature. No acute changes in the blood tests were seen. 

The patient underwent laparotomy in June 2024, with identification of a large intraluminal mass in the transverse colon, invading the head of the pancreas. Extended partial colectomy, lymphadenectomy, and appendectomy were performed. During the procedure, appendiceal diverticula were also identified as an incidental finding. The postoperative course was uneventful, with favorable evolution. He was discharged in good condition on the third day.

**Figure 3:**
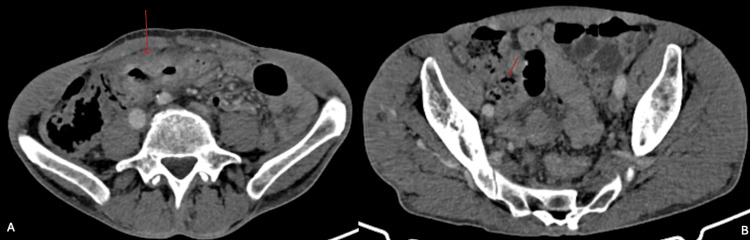
Abdominal CT - Case 2 Case 2. A: Tumor in the transverse colon. B: Appendix partially visualized. The CT reported circumferential parietal thickening in the transverse colon, close to the hepatic flexure, with luminal reduction of the colon. No alterations were reported in the appendix.

**Figure 4:**
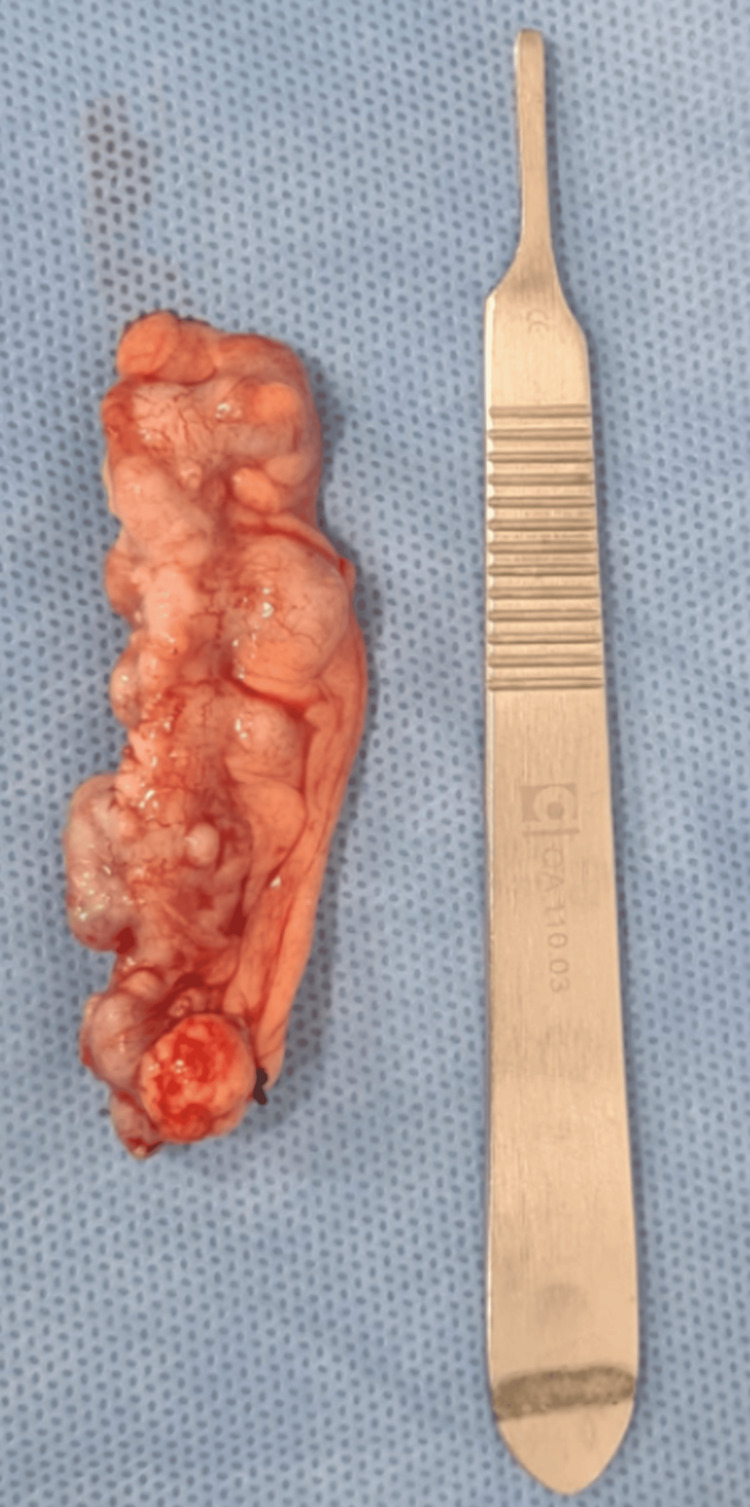
Appendectomy product - Case 2 Case 2. Diverticula consisting of all layers of the wall, with edema and vascular congestion, compatible with appendiceal diverticula.

Histopathological examination revealed moderately differentiated adenocarcinoma of the colon, with margins free of neoplasia and negative lymph nodes (pT3 pN0 pMx). The patient's abdominal CT is shown in Figure 3, with no report of diverticula, also diagnosing AD only after the biopsy result. Histological sections of the cecal appendix showed diverticula consisting of all layers of the wall, with edema and vascular congestion, compatible with appendiceal congenital diverticula, as shown in Figure 4.

Case 3: M.A.R.S

A 79-year-old female patient with a history of hypertension, hypothyroidism, and type 2 diabetes, without major abdominal surgeries, presented with abdominal pain and diarrhea for two days. She denied other symptoms. On physical examination, she had mild abdominal pain in the right iliac fossa, with no signs of peritoneal irritation.

On the same day, an abdominal CT scan was performed, which revealed an enlarged cecal appendix with slight densification of the adjacent adipose planes. She presented with leukocytosis and a slight increase in CRP in the laboratory review.

**Figure 5:**
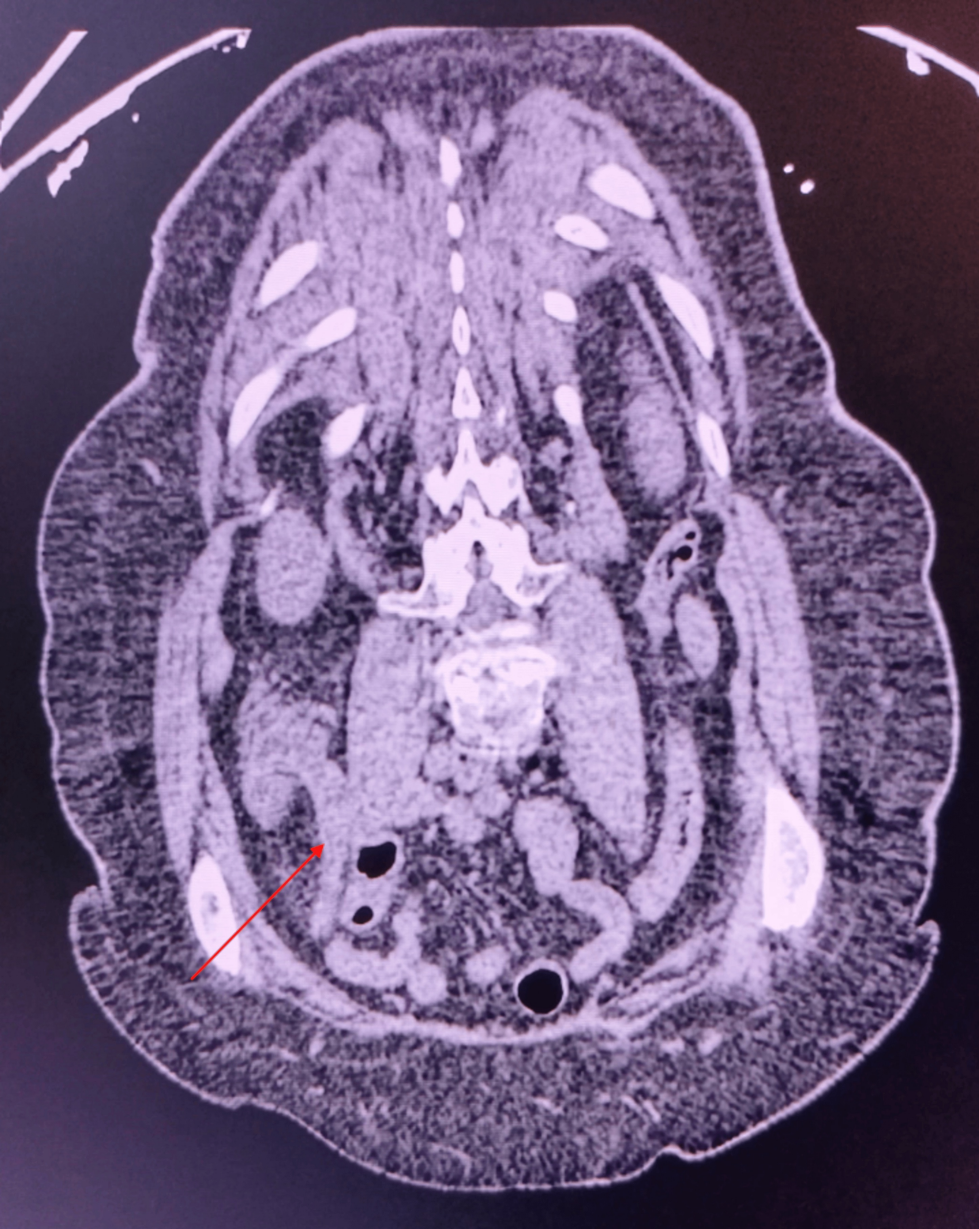
Abdominopelvic CT Case 3. Red arrow showing the inflamed appendix with slight densification of the adjacent adipose planes, without signs of diverticulitis.

**Figure 6:**
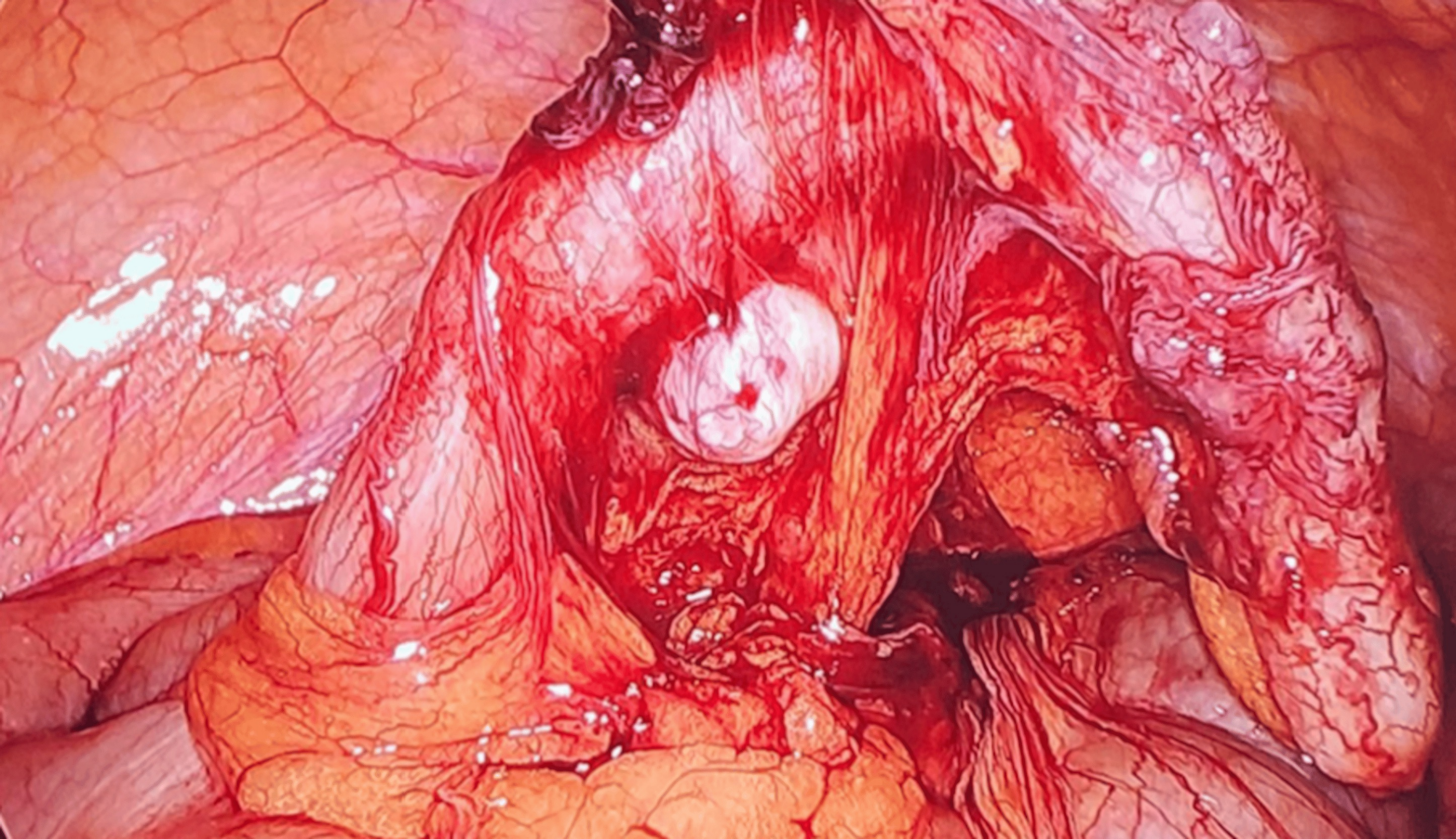
Videolaparoscopic appendectomy Case 3. Appendicular diverticulum.

**Figure 7:**
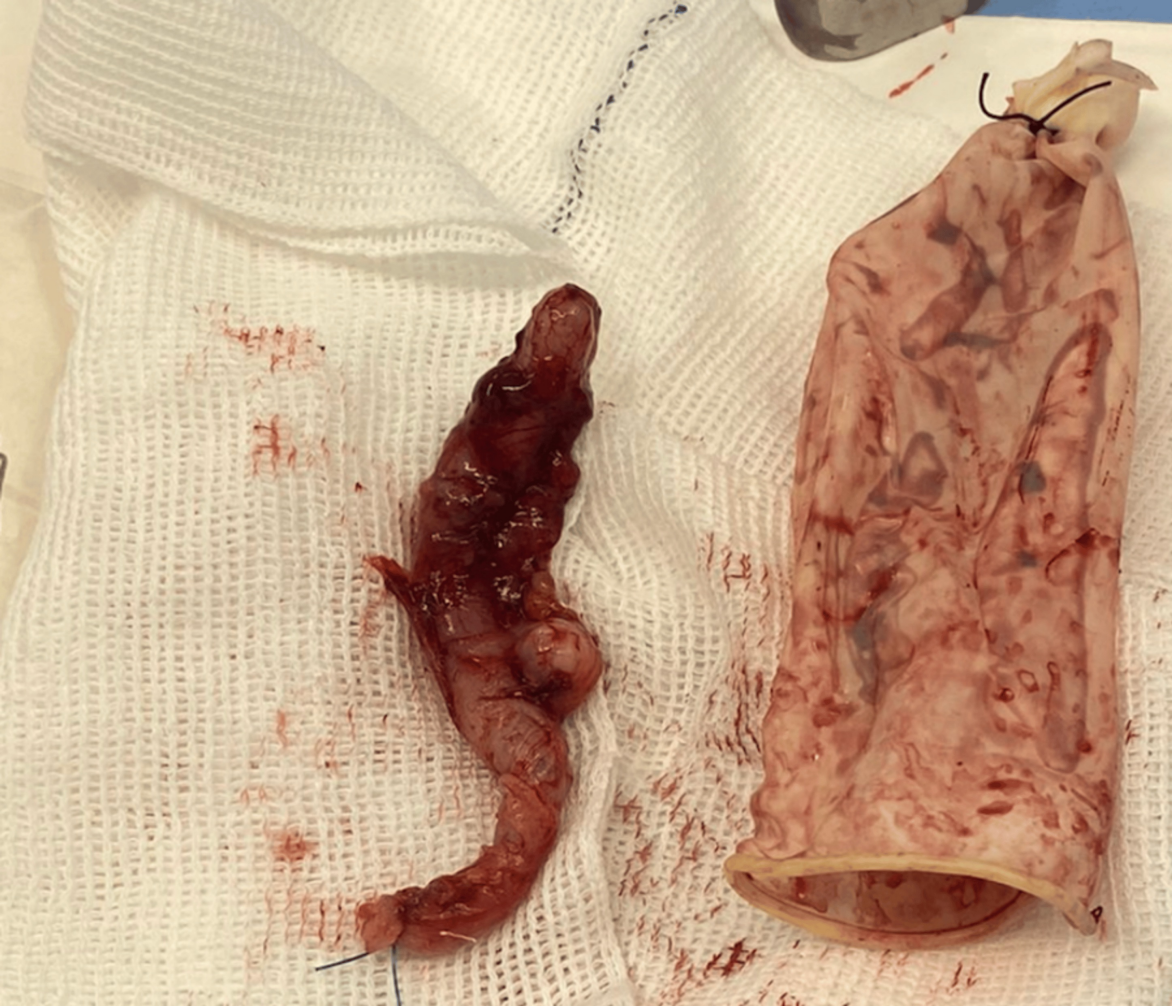
Appendectomy product - Case 3 Case 3. Acute appendicitis with periappendicitis and findings of associated appendiceal diverticulitis.

A videolaparoscopic appendectomy was performed without complications, and the patient was discharged on the first postoperative day in excellent condition. The anatomopathological examination revealed acute appendicitis with periappendicitis and findings of associated appendiceal diverticulitis. Next, we show the appendix in the abdominopelvic CT in Figure 5, during the procedure in Figure 6, and after its removal in Figure 7. The CT scan only reported an inflamed appendix, but without a report of diverticula, demonstrating the difficulty of pre-operative diagnosis. 

## Discussion

The Preferred Reporting Items for Systematic Reviews and Meta-Analyses (PRISMA) protocol was used to conduct the review in order to ensure methodological rigor. The Google Scholar, PubMed, and Scientific Electronic Library Online (SciELO) databases were used to search for publications between 1990 and 2024. The keywords used through the Health Science Descriptors (DeCS) and Medical Subjects Headings (MeSH) were appendicular diverticulum, appendix, appendectomy, and diverticular disease. The inclusion criteria were as follows: (1) articles available in Portuguese, English, or Spanish; (2) studies describing similar clinical and pathological characteristics, as well as diagnostic modalities and established treatment; (3) positive histopathological report for AD or some evidence of the disease; and (4) that they are case reports or case series. The exclusion criteria were as follows: (1) publications outside the stipulated period, (2) articles in other languages, and (3) diverticula in other regions.

In each case report analyzed, the following data were collected: author and year of publication; patient's data, such as age and sex; clinical presentation; type of surgery, histopathological result, and neoplastic association.

**Figure 8:**
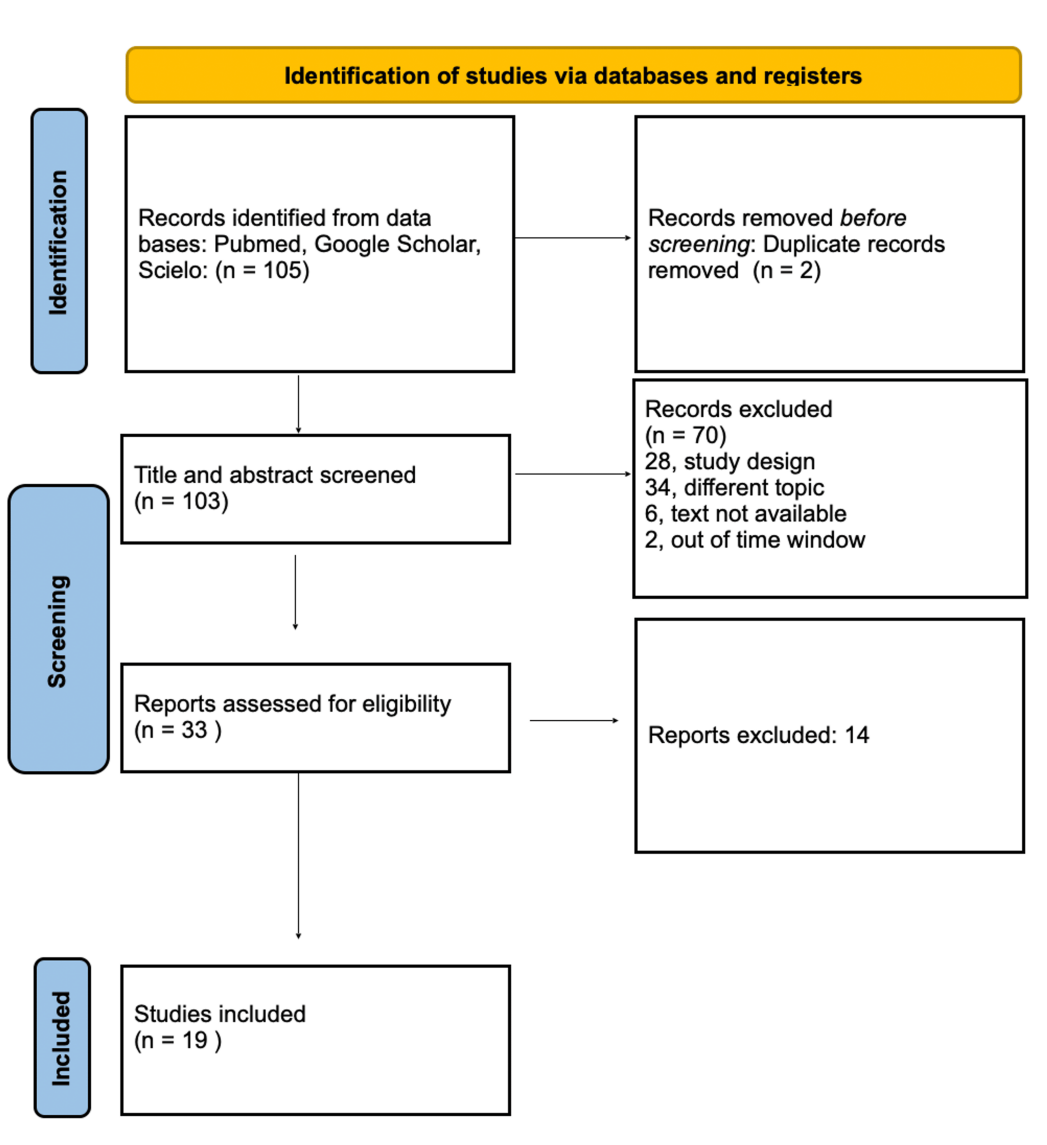
Preferred Reporting Items for Systematic Reviews and Meta-Analyses (PRISMA) flowchart

The literature search identified a total of 105 articles, two of which were excluded due to duplication. A total of 66 articles were analyzed in the PubMed database, 33 in Google Scholar, and one in SciELO. Subsequently, an initial screening was performed by reading the title and abstract. Of these, 70 were excluded for the following reasons: (1) study design (N = 28); (2) different theme (N = 34); (3) unavailable information (N = 6); and (4) articles outside the stipulated time period (N = 2). Thirty-three articles were then chosen for complete and detailed reading, with the intention of evaluating their inclusion or not. Finally, 19 studies were included, as seen in Figure 8 with the PRISMA flowchart and detailed in Table 1.

**Table 1:** Summary of the main findings of the analyzed studies on appendiceal diverticulum N.D. = not determined; US = ultrasonography

Authors/year	Title	Sex	Age	Clinical presentation/labs	Imaging	Treatment	Surgical findings	Histology	Outcome
Phillips et al. - (1999) [7]	Appendiceal diverticulitis	Masculine	59 years	Band-shaped abdominal pain, anorexia, and fever. Leukocytosis and neutrophilia.	CT	Videolaparoscopic appendectomy	Ruptured appendix	Appendicular diverticulitis	Postoperative period was uneventful.
Friedlich et al. - (2004) [8]	Diverticulitis of the appendix	Masculine	38 years	Right lower quadrant pain. His white blood cell count was elevated.	CT	Videolaparoscopic appendectomy	Appendicitis and also diverticular disease of the appendix.	N.D.	N.D.
Heffernan et al. - (2008) [9]	A case of appendiceal diverticulitis and a review of the literature	Feminine	46 years	Abdominal pain in the right hemi-abdomen, associated with tachycardia, fever, and leukocytosis of 16 × 10^9^/L).	CT	Laparotomy - extensive history of previous surgeries with adhesions - followed by appendectomy.	Perforated appendicitis	Perforated appendiceal diverticulum with abscess formation.	Postoperative period was uneventful.
Halder et al. - 2009 [10]	An Indian female presenting with appendicular diverticulitis: a case report and review of the literature	Feminine	29 years	Abdominal pain in the right iliac fossa, fever, anorexia and leukocytosis in labs.	N.D.	Appendectomy	Normal appendix with multiple diverticula	Appendicular diverticulitis	N.D.
Escobar et al. - (2013) [2]	Appendiceal diverticulitis, review of the scientific literature and presentation of two cases	Masculine	43 years	Pain in the epigastrium and irradiation to the left hemithorax, associated with constipation and abdominal distension, without signs of peritoneal irritation. Leukocytosis (12.9 × 10^9^/L).	N.D.	Appendectomy	Edematous appendix, with multiple diverticula measuring 3 to 7 mm in diameter. Purulent content in the lumen, middle, and distal third.	Appendicular diverticulosis with perforated diverticulitis.	The postoperative period was uneventful, and she was discharged on the fourth day.
Escobar et al. - (2013) [2]	Appendiceal diverticulitis, review of the scientific literature and presentation of two cases	Feminine	41 years	Abdominal pain in the epigastrium radiating to the right lower quadrant, signs of peritoneal irritation, and mass in the right iliac fossa.	N.D.	Appendectomy	Appendicular plastron, retrocecal appendix with firm adhesions and multiple diverticula, one of which was perforated, with pus leakage.	N.D.	The postoperative period was uneventful and she was discharged on the third day.
Zubieta-O'Farrill et al. - (2014) [11]	Appendiceal diverticulum associated with chronic appendicitis	Feminine	73 years	Moderate, oppressive pain for four months in the right lower quadrant of the abdomen, without radiation or any other symptoms.	CT	Videolaparoscopic appendectomy	Increased diameter of the appendix in its middle (1.3 cm) and distal (1.8 cm) thirds.	N.D.	The postoperative period was uneventful and she was discharged on the third day.
Motos Micó et al. - (2015) [12]	Appendiceal diverticulitis: a possible diagnosis in acute abdomen	Feminine	54 years	Abdominal pain initially localized in the right iliac fossa and fever. Positive Blumberg sign.	Abdominal US	Appendectomy	Appendix with 8 mm in diameter and thickening of periappendiceal fat and free fluid.	Diverticulosis of the vermiform appendix accompanied by mucocele and eosinophil-rich abscesses.	The postoperative period was uneventful, and she was discharged on the third day.
Altieri et al. - (2017) [13]	Appendiceal diverticulitis, a rare relevant pathology: presentation of a case report and review of the literature	Masculine	40 years	Right lower quadrant pain associated with vomiting, abdominal tenderness, fever, and moderate leukocytosis (11.93 × 10^9^/L; neutrophils 78.5%).	N.D.	Appendectomy	The removed appendix was 11 cm long and had multiple hyperemic and edematous diverticular protrusions.	N.D.	The postoperative period was uneventful. The patient was discharged on the 4th postoperative day.
Ng et al. - (2017) [14]	Appendiceal diverticulosis: a harbinger of underlying primary appendiceal adenocarcinoma?	Masculine	Middle-age adult	Pain in the right iliac fossa and anorexia. Mass in the iliac fossa on physical examination, leukocytosis, and neutrophilia.	CT	Videolaparoscopic appendectomy	Perforated appendix with periappendiceal abscess formation.	Perforated appendiceal diverticulitis and appendiceal adenocarcinoma T2N0Mx	Discharged in good clinical condition, patient subsequently declined hemicolectomy.
Albeeshi et al. - (2019) [15]	Appendiceal diverticulitis presenting as acute appendicitis diagnosed postoperatively	Feminine	28 years	Periumbilical pain, radiating to the right lower quadrant, associated with nausea, anorexia, and dysuria, without other urinary changes. There was no fever, weight loss, or change in bowel habits. The leukocyte count was 9.42 × 10^9^/L with a neutrophil differential of 62.3%.	CT	Videolaparoscopic appendectomy	Acute appendicitis.	N.D.	The patient was discharged without complications.
Hwala et al. - (2019) [16]	Appendiceal diverticulitis presenting with clinical features of acute appendicitis	Masculine	44 years	Periumbilical pain, leukocytosis, and neutrophilia.	CT	Videolaparoscopic appendectomy and Blake drain	Inflamed appendix and saccular structure near the base.	Appendicular diverticulitis	N.D.
Fiordaliso et al. - (2020) [17]	A case of type 2 appendiceal diverticulum perforated and a review of the literature	Masculine	68 years	Progressive band-like abdominal pain associated with constipation and fever. The leukocyte count was 14 × 10^9^/L.	CT	Videolaparoscopic appendectomy	Dilated appendix, thickening of the appendiceal wall with enhancement, free periappendiceal fluid	A single appendiceal diverticulum was identified, with acute inflammation and perforation.	The patient was discharged without complications.
Liu et al. - (2021) [18]	Perforated appendiceal diverticulitis mimicking appendicitis	Feminine	57 years	Nine days of centralized abdominal pain, progressing to the iliac fossa. Initially normal tests.	CT	Laparoscopic appendectomy and cecectomy	Marked thickening of the appendix and dilated (1.6 cm). Dilated appendix with suspected neoplasia	Ruptured appendiceal diverticulum with leaking mucus	The patient was discharged without complications.
Onafowokan et al. - 2021 [19]	Appendiceal diverticulitis in a young female diagnosed on pathology after laparoscopic appendectomy	Feminine	23 years	Abdominal pain in the right iliac fossa and nausea.	CT	Videolaparoscopic appendectomy	Dilated, inflamed, thick-walled appendix.	Appendix with inflamed diverticulum	The patient was discharged without complications.
Bujold-Pitre et al. - (2021) [20]	Diverticulitis of the appendix—case report and literature review	Masculine	72 years	Abdominal pain localized to the left upper quadrant. Total white blood cell count was 13.0 × 10^9^/L.	CT	Diagnostic laparoscopy - due to the suspected neoplasia and the anatomical characteristics, right hemicolectomy with primary anastomosis was chosen.	Enlarged and edematous appendix with periappendiceal fat blurring compatible with acute appendicitis.	Appendicitis with multiple inflamed appendiceal diverticula. No neoplasms were identified.	The patient was discharged without complications.
Abdulmomen et al. - (2022) [21]	Acute perforated appendicitis associated with appendiceal diverticulitis in a young man: a case report with literature review	Masculine	35 years	Abdominal pain in the right lower quadrant that radiated to the left lower quadrant, associated with fever, vomiting and abdominal distension. Biochemical analysis revealed mild leukocytosis (11 × 10^9^/L).	CT	Videolaparoscopic appendectomy	Acute perforated appendicitis. The body of the appendix was dilated, with at least two wall defects associated with small loculations of fluid. Thickening of the periappendiceal fat and enhancement of the adjacent peritoneal lining, suggesting focal peritonitis.	N.D.	On the third postoperative day, the patient was discharged in good condition.
Drew et al. - (2022) [22]	Complicated appendicular diverticulitis	Masculine	75 years	Periumbilical and right iliac fossa pain, with tenderness at McBurney's point and elevated white blood cell count (15.2 × 10^9^/L).	CT	Appendectomy	A 15 mm thin-walled diverticulum arising from the tip of the appendix, collection of fluid containing gas, indicating perforation. The appendix was dilated (13 mm), with thickened and hyperenhanced walls.	N.D.	N.D.
Bonomo et al. - (2022) [23]	Surgical rarities: case report of appendicular diverticulitis and literature review	Masculine	22 years	Pain in the right iliac fossa had begun the previous day. Physical examination showed tenderness at McBurney's point. There was no report of fever or vomiting. Blood tests indicated only a mild increase in C-reactive protein (0.71 mg/dL).	Abdominal US	Videolaparoscopic appendectomy	Appendix with a diameter of 10 mm, thickened walls, rounded image filled with fluid at the distal tip, and minimal collection of periappendiceal fluid.	N.D.	The postoperative course was uneventful and the patient was discharged on the third postoperative day.

**Figure 9:**
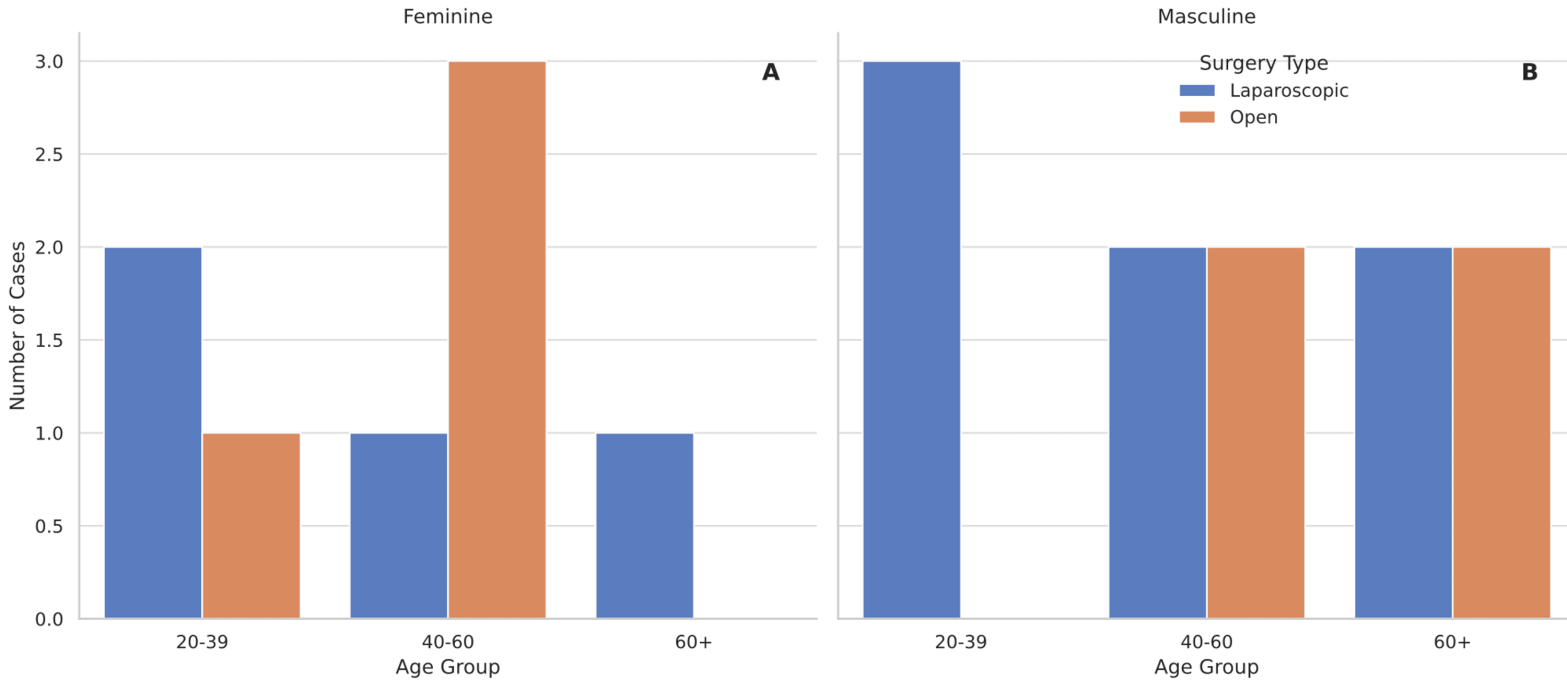
Distribution of surgical approach by age and sex in reported cases of appendiceal diverticulum A: Number of feminine cases. B: Number of masculine cases. Distribution of surgical approaches (laparoscopic vs. open) in 19 published cases of appendiceal diverticulum, categorized by sex and age group.

Figure 9 illustrates some important variables for better understanding the demographics of this disease in men and women.

Of the 19 patients with AD, 58% were male and 42% female, with most patients aged 40-60. Acute abdominal pain was the most common symptom among patients, specifically in the right lower quadrant. The primary diagnostic imaging method was abdominal CT.

More than half (63%) of the cases were managed laparoscopically, and the remainder underwent open surgery. In 27% of the patients, the appendix was perforated, and in one patient, appendiceal adenocarcinoma was found. All patients were discharged without major complications.

AD, although a rare entity, remains an important differential diagnosis. First described in 1893 by Kelynack, it has an incidence of approximately 0.0014 - 2% of cases - with the literature reporting between 1.74% [6], 0.6% [24], 0.59% [25], and 2.6% [26,27]. A large study of 71,000 appendectomies reported appendiceal diverticulosis in 0.5-1.5% of cases [28].

The disease is classified into two histological types: congenital and acquired. The congenital type is a true diverticulum, with three layers (mucosa, submucosa, muscularis, and serosa) and is usually located on the antimesenteric border of the appendix. The mechanisms involved are related to failure in the recanalization of the appendiceal lumen, obliteration of the vitelline duct, duplication of the appendix, or syndromes such as Patau syndrome [13].

The acquired type is characterized by being a small pseudodiverticulum (mucosa and submucosa), located in the distal region of the appendix on the mesenteric border. The etiology is not known, but there are some risk factors such as male sex, adult age, and cystic fibrosis, among others. The pathogenesis may be related to recurrent inflammatory or infectious episodes that extend the lumen and generate lymphoid atrophy, which ends up creating a weakness in the wall and consequently the diverticulum (and perforation). Another theory, non-inflammatory, is related to obstruction of the lumen by some cause (fecalitis, tumor, etc.) that increases muscle contraction [13,29].

There are several classifications of AD, but probably the most widely used is that of Phillips et al., where type 1 is acute primary diverticulitis with or without peri-diverticulitis; type 2 is diverticulitis secondary to appendicitis; type 3 is uncomplicated diverticulum; type 4 is diverticulum with acute appendicitis; and type is 5 chronic peridiverticulitis with acute appendicitis [7].

In AD, the patient is usually asymptomatic, and when diverticulitis is present, it is difficult to diagnose preoperatively and is usually misdiagnosed as appendicitis. It is characterized by abdominal pain in the right lower quadrant, a milder and more insidious pain lasting approximately seven to 14 days, compared to the acute pain of appendicitis [2,9]. Other important data to consider at the time of diagnosis are male gender and older age (>30 years), which are also risk factors [30]. In the study by Yamana et al., 12 patients with AD were included, 10 men and two women, with a mean age of 42.7 years [5]. In this study, these factors were evident, with most patients being adults and men.

For diagnosis, US can be used, which can have a specificity of up to 100%, but always depending on the experience of the radiologist, and CT, where a protrusion in the appendix is observed. Inflamed diverticula have thickened walls or a solid mass with a lumen filled with air or fluid. Osada et al. reported preoperative appendiceal diverticulosis on CT in six of 156 patients who underwent appendectomy [23,31]. In another case series, abdominal CT was conducted in 34 patients with appendiceal diverticulitis, none of whom were diagnosed radiologically [25], so it is not easy to diagnose AD preoperatively. In the current study, only two patients underwent US, and the rest underwent CT.

The importance of this disease lies in the complications it entails, which are perforation and neoplasia.

A study of 2,711 patients undergoing appendectomy demonstrated that patients with AD are 10 times more likely to suffer from neoplasia than those who do not suffer from the disease (17.5 vs. 1.8%; p < 0.0001, OR 11.8 95%, CI 5.6-24.8) [32]. In the study by Dupre et al., 11 cases of neoplasia were reported in a series of 23 patients with appendiceal diverticula (N = 1,361) (47.8% of all patients with AD) (p < 0.0001, Fisher's test) [33]. There is an association between AD and adenocarcinoma, carcinoid tumors, and mucinous neoplasms, the latter of which can result in a rare and serious complication, pseudomyxoma peritonei. In a study of 32 patients, 25% of cases contained a mucinous neoplasm [34]. A systematic review indicated that patients with appendiceal diverticulum have a prevalence rate of appendiceal neoplasm of up to 26.94%, compared with a rate of 1.28% in patients without diverticulum [6]. In our study, this did not occur, with only one patient diagnosed with appendiceal adenocarcinoma.

Perforation is another important complication of AD. The rate of perforation in AD varies from 30% to 70%. In the study by Sohn et al., it was shown that appendiceal perforation was found in 65.8% of the group with diverticulitis [29]. Yamana et al. reported a perforation rate of 33% [5]. Other bibliographies reported an incidence of up to 66% (four times higher than in acute appendicitis) with a mortality rate 30% higher when compared to acute appendicitis [25]. Among the cases in this study, there was an incidence of perforation compatible with the bibliography (27%).

The risk of perforation in acquired diverticulosis is higher than in congenital diverticulosis (up to 66% vs. 6.6%, respectively) due to the thin wall and the absence of a thick muscularis propria [21].

Treatment will depend on the characteristics of the cecal appendix, but early open or laparoscopic appendectomy is indicated for appendiceal diverticulitis, and resection is under discussion for asymptomatic diverticulosis as found intraoperatively, due to the possible risk of perforation and neoplasia [21,32]. In the present study, two appendices were resected “preemptively” when laparotomies were being performed for another procedure. The remainder were for diverticulitis.

For oncologic cases, right hemicolectomy is generally advocated as the preferred intervention because of possible lymph node involvement in more than half of the cases. In a study of 94 patients diagnosed with adenocarcinoma of the appendix, the five-year survival rate was 73% in the right hemicolectomy group compared with 44% in the appendectomy group [14,35]. Frozen section is recommended in cases of high suspicion of malignancy. A diagnosis of T2 adenocarcinoma and above is an indication for right hemicolectomy in the same procedure [14]. In the case of diverticular adenocarcinoma, in this case, appendectomy alone was performed, and hemicolectomy was planned at a later stage, but the patient refused.

## Conclusions

Appendiceal diverticulosis, though rare, carries a significantly higher risk of perforation, abscess formation, and neoplastic transformation compared to typical acute appendicitis. In our case series and literature review, perforation occurred in 26% of patients (five out of 19) - underscoring the elevated risk associated with this condition. Clinically, appendiceal diverticulitis can be difficult to distinguish from acute appendicitis but should be suspected in middle-aged patients presenting with prolonged or atypical right lower quadrant pain, mild systemic signs, or a history of recurrent similar episodes. On CT imaging, focal outpouchings of the appendix and minimal wall thickening may suggest the diagnosis, particularly when symptoms seem disproportionate to imaging findings.

Early recognition, whether through imaging or intraoperative assessment, is crucial. Given the increased risk of complications and the established association with appendiceal neoplasms, prophylactic appendectomy should be strongly considered, even in asymptomatic patients where diverticulosis is discovered incidentally during colorectal surgery or imaging. Current evidence is limited by small sample sizes and the retrospective nature of most available studies, including our own. As such, these findings should be interpreted with caution, and treatment decisions must be individualized. Further prospective studies are necessary to better define the oncologic implications and to establish standardized management strategies for both symptomatic and incidental cases of appendiceal diverticulosis.

## References

[1] Çakar E, Bayrak S, Çolak Ş (2019). Clinical characteristics of appendiceal diverticular disease. Int J Colorectal Dis.

[2] Escobar F, Vega N, Valbuena E (2013). Appendiceal diverticulitis, review of the scientific literature and presentation of two cases. Rev Colomb Cir.

[3] Sugihara K, Muto T, Morioka Y, Asano A, Yamamoto T (1984). Diverticular disease of the colon in Japan. A review of 615 cases. Dis Colon Rectum.

[4] Tursi A, Scarpignato C, Strate LL, Lanas A, Kruis W, Lahat A, Danese S (2020). Colonic diverticular disease. Nat Rev Dis Primers.

[5] Yamana I, Kawamoto S, Inada K, Nagao S, Yoshida T, Yamashita Y (2012). Clinical characteristics of 12 cases of appendiceal diverticulitis: a comparison with 378 cases of acute appendicitis. Surg Today.

[6] Lim CS, Cheah SY, Kwok AM, Ravindran P, Chan DL (2020). Systematic review and meta-analysis of the association between diverticulosis of the appendix and neoplasia. ANZ J Surg.

[7] Phillips BJ, Perry CW (1999). Appendiceal diverticulitis. Mayo Clin Proc.

[8] Friedlich M, Malik N, Lecompte M, Ayroud Y (2004). Diverticulitis of the appendix. Can J Surg.

[9] Heffernan DS, Saqib N, Terry M (2009). A case of appendiceal diverticulitis, and a review of the literature. Ir J Med Sci.

[10] Halder SK, Khan I (2009). An Indian female presenting with appendicular diverticulitis: a case report and review of the literature. Cases J.

[11] Zubieta-O'Farrill G, Guerra-Mora JR, Gudiño-Chávez A, Gonzalez-Alvarado C, Cornejo-López GB, Villanueva-Sáenz E (2014). Appendiceal diverticulum associated with chronic appendicitis. Int J Surg Case Rep.

[12] Motos Micó J, Ferrer Márquez M, Berenguel Ibáñez MM, Belda Lozano R, Moreno Serrano (2015). Appendiceal diverticulitis: a possible diagnosis in acute abdomen. Cir Esp.

[13] Altieri ML, Piozzi GN, Salvatori P, Mirra M, Piccolo G, Olivari N (2017). Appendiceal diverticulitis, a rare relevant pathology: presentation of a case report and review of the literature. Int J Surg Case Rep.

[14] Ng JL, Wong SL, Mathew R (2018). Appendiceal diverticulosis: a harbinger of underlying primary appendiceal adenocarcinoma?. J Gastrointest Oncol.

[15] Albeeshi MZ, Alwanyan AA, Salim AA, Albabtain IT (2019). Appendiceal diverticulitis presenting as acute appendicitis diagnosed postoperatively. J Surg Case Rep.

[16] Hwala S., Aoun C., El Hajj I. (2019). Appendiceal diverticulitis presenting with clinical features of acute appendicitis: case report and literature review. World J Surg Surgical Res.

[17] Fiordaliso M, De Marco AF, Costantini R (2020). A case of type 2 appendiceal diverticulum perforated and a review of the literature. Int J Surg Case Rep.

[18] Liu Liu, K. K. (2025). Perforated appendiceal diverticulitis mimicking appendicitis. American college of surgeons.

[19] Onafowokan OO, Khairat A, Bonatti HJ (2021). Appendiceal diverticulitis in a young female diagnosed on pathology after laparoscopic appendectomy for acute appendicitis. Case Rep Med.

[20] Bujold-Pitre K, Mailloux O (2021). Diverticulitis of the appendix-case report and literature review. J Surg Case Rep.

[21] Abdulmomen AA, AlZahrani AS, Al Mulla LA, Alaqeel FO (2022). Acute perforated appendicitis associated with appendiceal diverticulitis in a young man: a case report with literature review. Am J Case Rep.

[22] Drew ZJ, Chakrabarty S, Malghan R (2022). Complicated appendicular diverticulitis. J Med Radiat Sci.

[23] Bonomo LD, Zago M, Quirico C (2022). Surgical rarities: case report of appendicular diverticulitis and literature review. J Surg Case Rep.

[24] Ergenç M, Uprak TK (2022). Appendiceal diverticulitis presenting as acute appendicitis and diagnosed after appendectomy. Cureus.

[25] Marcacuzco AA, Manrique A, Calvo J (2016). Clinical implications of diverticular disease of the appendix: experience over the past 10 years. Cir Esp.

[26] Bianchi A, Heredia A, Hidalgo LA, García-Cuyàs F, Soler MT, del Bas M, Suñol X (2005). Diverticular disease of the cecal appendix. Cir Esp.

[27] Muñoz CC, Mansilla EJ, Roa SC, Heider CC (2011). Prevalence of diverticular disease of the cecal appendix in patients appendectomized for acute appendicitis. Rev Chil Cir.

[28] Collins DC (1963). 71,000 Human appendix specimens. A final report, summarizing forty years’ study. Am J Proctol.

[29] Sohn TJ, Chang YS, Kang JH (2013). Clinical characteristics of acute appendiceal diverticulitis. J Korean Surg Soc.

[30] Lesi OK, Probert S, Iqbal MR (2022). Diverticulitis and diverticulosis of the appendix: a case series. Cureus.

[31] Osada H, Ohno H, Saiga K, Watanabe W, Okada T, Honda N (2012). Appendiceal diverticulitis: multidetector CT features. Jpn J Radiol.

[32] Chan DL, Lim C, Bakhtiar A, Khoury M, Smigelski M, Yeh D, Ravindran P (2018). Clinical significance of appendiceal diverticulum: a significant marker for appendiceal neoplasia in Australian patients. Int J Colorectal Dis.

[33] Dupre MP, Jadavji I, Matshes E, Urbanski SJ (2008). Diverticular disease of the vermiform appendix: a diagnostic clue to underlying appendiceal neoplasm. Hum Pathol.

[34] Lamps LW, Gray GF Jr, Dilday BR, Washington MK (2000). The coexistence of low-grade mucinous neoplasms of the appendix and appendiceal diverticula: a possible role in the pathogenesis of pseudomyxoma peritonei. Mod Pathol.

[35] Nitecki SS, Wolff BG, Schlinkert R, Sarr MG (1994). The natural history of surgically treated primary adenocarcinoma of the appendix. Ann Surg.

